# Prevalence of HPV infections in surgical smoke exposed gynecologists

**DOI:** 10.1007/s00420-020-01568-9

**Published:** 2020-09-01

**Authors:** Xiaoli Hu, Qingfeng Zhou, Jian Yu, Jing Wang, Quanmei Tu, Xueqiong Zhu

**Affiliations:** Department of Obstetrics and Gynecology, The Second Affiliated Hospital of Wenzhou Medical University, No. 109 Xueyuan Xi Road, Wenzhou, 325027 Zhejiang China

**Keywords:** Electrosurgery, Human papillomavirus, Surgical smoke, Loop electrosurgical excision procedure

## Abstract

**Objectives:**

Human papillomavirus (HPV) has been reported recently in surgical smoke generated by gynecological operations. The objective of this study was to investigate whether gynecologists who have performed electrosurgery including loop electrosurgical excision procedure (LEEP), are at risk of acquiring HPV DNA through surgical smoke.

**Methods:**

A related questionnaire was designed and 700 gynecologist nasal swab samples were collected in 67 hospitals. In addition, the flow fluorescence hybridization technique was used to detect HPV DNA, and the Chi-square test was applied to analyze whether related risk factors including electrical surgery, were correlated with HPV infection in surgeons’ nasal epithelial cells.

**Results:**

The HPV infection rate in the nasal epithelial cells of the participants who performed electrosurgery (8.96%, 42/469) or LEEP (10.11%, 36/356) was significantly higher than that in the remaining participants who did not perform electrosurgery (1.73%, 4/231) or LEEP (2.91%, 10/344), respectively. The most prevalent HPV genotype in the electrosurgery group was HPV16 (76.19%, 32/42). The HPV-positive rate was increased in the group that had a longer duration of electrosurgery (*P* = 0.016). Additionally, the HPV detection rate was significantly lower in electrosurgery operators who used surgical mask (7.64%, 33/432) than in those who did not use protective masks (24.32%, 9/37). Furthermore, the N95 mask (0%, 0/196) significantly reduced the risk for HPV infection compared to that with the general mask (13.98%, 33/236, *P* < 0.001). Furthermore, 46 participants infected with HPV were followed-up for 3–24 months, and approximately 43.48% (20/46) and 100% (41/41) became negative for HPV DNA, respectively.

**Conclusions:**

Gynecologists who performed electrosurgery including LEEP were at risk of acquiring HPV infection. Surgical masks, especially the N95 mask, significantly decreased the hazard of HPV transmission from surgical smoke.

**Electronic supplementary material:**

The online version of this article (10.1007/s00420-020-01568-9) contains supplementary material, which is available to authorized users.

## Introduction

Cervical cancer is the third most common gynecological cancer worldwide and one of the main reasons for cancer-related death in females in developing countries (Siegel et al. 2018). The presence of high-risk types of human papillomavirus (HPV) infection, especially HPV types 16 and 18, plays a crucial role in cervical lesions and even cervical carcinoma (Ainouze et al. 2018; Liu et al. 2018). Currently, electrical operation, including laser ablation and electrocauterization, are widely used to treat a variety of cervical lesions. Among such procedures, cervical cauterization including the loop electrosurgical excision procedure (LEEP) has rapidly become one of the most widely available techniques used to excise cervical biopsies. In recent studies, guidelines that provide recommendations for the use of LEEP to treat histologically confirmed cervical intraepithelial neoplasia (CIN) II–III have been described, along with recommendations for screening and treatment strategies (Papasavvas et al. 2018; Santesso et al. 2016). Electrical operations including LEEP which uses low-voltage, high-frequency radio waves through a thin wire loop to accomplish surgical effects may cause tissue destruction and may liberate intact cells and including viral DNA, which may be a potential risk to surgeons (Sood et al. 1994).

Surgical smoke can be produced during electrocautery or ultrasonic scalpel procedures. Surgical smoke is the gaseous by-product arising from tissue being burned, dissected or cauterized by heat generating devices such as laser surgery or diathermy (Georgesen and Lipner 2018; Sanderson 2012), which may be a biohazard to surgeons (Choi et al. 2014). Recently, the possibility that surgeons might inhale viral particles such as HPV DNA from surgical smoke during the removal of certain lesions has been pointed out by several authors (Gloster and Roenigk 1995).

Surgical masks are an important measure used for protecting surgeons from surgical plumes. Traditional surgical masks filter out particulates only as small as 5 μm in diameter, and even high-filtration masks can filter particles larger than approximately 0.1 μm in size (Benson et al. 2013; Coleman 2014). In reality, the particulate HPV virus can range in size from 45 to 55 nm. As a result, most surgical masks even high-filtration masks do not have adequate filtering or appropriate attributes to provide respiratory protection for wearers, and the particulate of viruses ranging from 0.01 to 0.3 μm in size can be trapped in the airways and can penetrate directly into the trachea, bronchioles and alveoli (Walczak et al. 2011).

Smoke evacuation is another effective measure which that has the ability to capture the smoke generated at the surgical site and remove it to an area away from the surgical team (Szekrenyesi et al. 2018). A recent survey revealed that effective smoke evacuation, such as local exhaust ventilation procedures and central plume evacuation systems, was used by fewer than half of the facilities for most laser procedures, electrosurgery and diathermy procedures (Ulmer 2008). Thus, surgeons encounter more potential health risks. However, most surgeons, perioperative personnel, and health care organizations lack general knowledge regarding the potential health risks associated with exposure to surgical smoke and underuse measures that may offer effective self-protection. Both the outpatient office setting where most LEEPs are performed in China and the operating room have a paucity of protective facilities to protect gynecologists from smoke exposure.

Thus, the aim of the present study was to explore the prevalence of HPV in nasal swabs from clinicians exposed to surgical smoke.

## Materials and methods

### Questionnaire survey

A questionnaire was designed and administered to gynecologists in various hospitals. The self-administered questionnaire, which included items to evaluate the transmission of HPV DNA from patients to gynecologists, consisted of several questions, such as whether the electrical operation included LEEP, other electrocautery or laser ablation; HPV vaccine status; total operation time; and protective measures. As shown in SI 1, the questionnaire was verified by a study coordinator (Zhou Q.F.) who was on the scene when medical workers were canvassed through the self-administered questionnaire. Any uncertain items were queried by a quality control engineer (Tu Q.M.) by telephone.

### Sample collection

All the medical personnel voluntarily participated in the research and signed a written consent form. Participants were asked to provide nasal swab samples for DNA extraction and HPV detection. The nasal swab samples were collected by Zhou Q.F. and obtained from both sides of the operators’ nasal cavity with a depth of 2–3 cm using a sterile cotton swab which was then inserted into a 1.0 mL of phosphate buffered saline (PBS). The collected samples were subjected to HPV genotype detection by sequencing. All specimens were stored at − 20 °C immediately after collection.

### DNA extraction

A high-purity viral nucleic acid kit (Roche, Mannheim, Germany) was applied to extract HPV DNA from human nasal epithelial cells. A total of 200 μL of digestion buffer was added to the sample solution. After incubation at 72 °C for 10 min, 100 μL of binding buffer was added and mixed immediately. After centrifugation, 500 μL of inhibitor removal buffer was added to the supernatant. The buffer was centrifuged at 8000 rpm for 1 min. The filter tube was inserted into a nuclease-free tube, and 50 μL of elution buffer was eluted in the purified DNA. Afterwards, the DNA was resuspended and stored at − 20 °C until needed (Ji et al. 2018).

### DNA sample quality assessment

The Protein Nucleic Acid Analyzer (Thermo Scientific, Shanghai, China) was applied to evaluate the quality of the DNA extracted by measuring the optical density (OD) of absorbance OD260/OD280 which followed the manufacturer’s recommendations. Additionally, the human β-globin gene from the collected specimen was amplified to monitor the quality of the DNA samples for subsequent DNA amplification.

### HPV genotyping by hybridization

HPV DNA detection was performed using the Tellgenplex™ HPV DNA Test (Tellgen, Shanghai, China) according to the manufacturer’s instructions. Genotyping was carried out by a flow-through hybridization technique using HPV DNA amplified by polymerase chain reaction (PCR). This testing method can detect 27 HPV genotypes, including 17 high-risk HPV types (HPV16, 18, 26, 31, 33, 35, 39, 45, 51, 52, 53, 56, 58, 59, 66, 68 and 82), and 10 low-risk HPV types (HPV6, 11, 40, 42, 43, 44, 55, 61, 81 and 83). Appropriate positive and negative controls were used in all PCR amplifications. The PCR system volume was 20 μL, including 5 μL of primer mixture, 9.2 μL of PCR premixed solution, 0.8 μL of Taq DNA polymerase and 5 µL of extracted DNA. In total, 40 cycles were performed (Rogeri et al. 2018).

The fluorescence hybridization method was performed on an instrument at 45 °C, and 22 µL of hybridization solution and 3 µL of PCR product were added into 96-well microtiter plates (Tellgen, Shanghai, China) for each well. The negative control assay was performed at the same time. The product was detected by a Luminex 200 analyzer instrument (Luminex Corporation, Austin, TX). The HPV-genotype result was determined according to the position of the HPV-genotype probes on the microarray chip. Multiple dots indicate multiple infections.

### Follow-up studies

Surgeons whose nasal swab specimens tested positive for HPV DNA received additional HPV DNA tests for nasal swabs at 3, 12 and 24 months.

### Statistical analysis

Statistical analysis was performed using SPSS 17.0 statistical software (SPSS, Chicago, IL, USA). Age is expressed as the mean and standard deviation (SD). A Chi-square test was conducted to identify the significance of differences between two groups. A logistic regression model was used to analyze the correlation between risk factors and HPV infection among doctors performing electrocardiosurgery of the cervix. There was statistical significance when *P *< 0.05.

## Results

### Study population

A total of 700 gynecologists in 67 local hospitals throughout Zhejiang Province from January 2016 to December 2016 were recruited to the study for detection of HPV DNA by HybriMax; their mean age was 35.1 ± 8.8 years (ranging from 21 to 72). The included hospitals comprised 25 third-grade class-A hospitals and 42 township hospitals. In addition, the relevant hospitals included Women’s Hospital, School of Medicine, Zhejiang University; Zhejiang Provincial People’s Hospital; the First Affiliated Hospital of Wenzhou Medical University; the Second Affiliated Hospital of Wenzhou Medical University; Taizhou Hospital of Zhejiang Province; the Central Hospital of Lishui City; and others.

### Participants’ characteristics

As shown in Table 1, the average age of the participants, including chief physicians, associate chief physicians, attending doctors and resident doctors, was 35.1 years with a standard deviation of 8.8 years and a range of 21–72 years. Approximately 16.43% of the 700 gynecologists were male, and the remaining 83.57% were female. A majority of participants (469/700, 67.00%) had conducted electrosurgery on the cervix. Among them, 356 doctors had performed LEEP, and 113 gynecologists had performed other electric surgeries, such as laser ablation or other electrocautery, rather than LEEP. Additionally, none of the doctors included in this study had received the HPV vaccine before our investigation.

**Table 1:** The basic demographics of respondents

Demographic	Total	Percentage (%)
Age (years)	700	
21–29	188	26.86
30–39	331	47.29
40–49	138	19.71
≥ 50	43	6.14
Gender	700	
Male	115	16.43
Female	585	83.57
Operation of electrosurgery	700	
Yes	469	67
No	231	33
Operation of LEEP in electrosurgery group	469	
Yes	356	75.91
No	113	24.09

### The HPV detection rate among the participants by electrosurgery type

Among all the gynecologists, the prevalence of HPV was 6.57% (46/700), with 6.43% (45/700) of the participants having a single viral type and 0.14% (1/700) having multiple viral types. As shown in Table 2, approximately 8.96% (42/469) of participants who had performed electrosurgery of the cervix were positive for HPV DNA, and 1.73% (4/231) of participants who had not performed electrosurgery were positive for HPV DNA. Based on statistical Chi-square analyses, it turned out that participants who had performed electrosurgery of the cervix had greater odds of acquiring HPV than participants who had not performed electrosurgery. The difference in HPV-positive rates between the two groups was statistically significant (*P* < 0.001).

**Table 2:** The correlation of positive rates in gynecologists who performed electrosurgery or not

	HPV testing		Total	*χ*^2^	*P* value
	Positive	Negative			
Electrosurgery	42 (8.96%)	427 (91.04%)	469		
No electrosurgery	4 (1.73%)	227 (98.27%)	231		
Total	46 (6.57%)	654 (93.43%)	700	13.154	0.000

Additionally, the overall rate of HPV positivity among operators performing LEEP was further evaluated. As shown in Table 3, the rate of HPV positivity in the included gynecologists who had performed LEEP was 10.11% (36/356), which was significantly higher than that in those who did not perform LEEP (2.91%, 10/344).

**Table 3:** The correlation of HPV-positive rates in gynecologists who performed LEEP or not

	HPV testing		Total	*χ*^2^	*P* value
	Positive	Negative			
LEEP	36 (10.11%)	320 (89.89%)	356		
No LEEP	10 (2.91%)	334 (97.09%)	344		
Total	46 (6.57%)	654 (93.43%)	700	13.154	0.000

The difference in the HPV-positive rates between the LEEP group and the other electrosurgery group was further investigated. As shown in Table 4, there was no significant difference (*P *= 0.119) between the two groups.

**Table 4:** The correlation of HPV-positive rates in gynecologists who performed LEEP or other electrosurgery of cervix such as laser ablation or other electrocauterization

	HPV testing		Total	*χ*^2^	*P* value
	Positive	Negative			
LEEP	36 (10.11%)	320 (89.89%)	356		
Other electrosurgery	6 (5.31%)	107 (94.69%)	113		
Total	42	427	469	2.427	0.119

### HPV genotypic distributions among participants

**Fig. 1:**
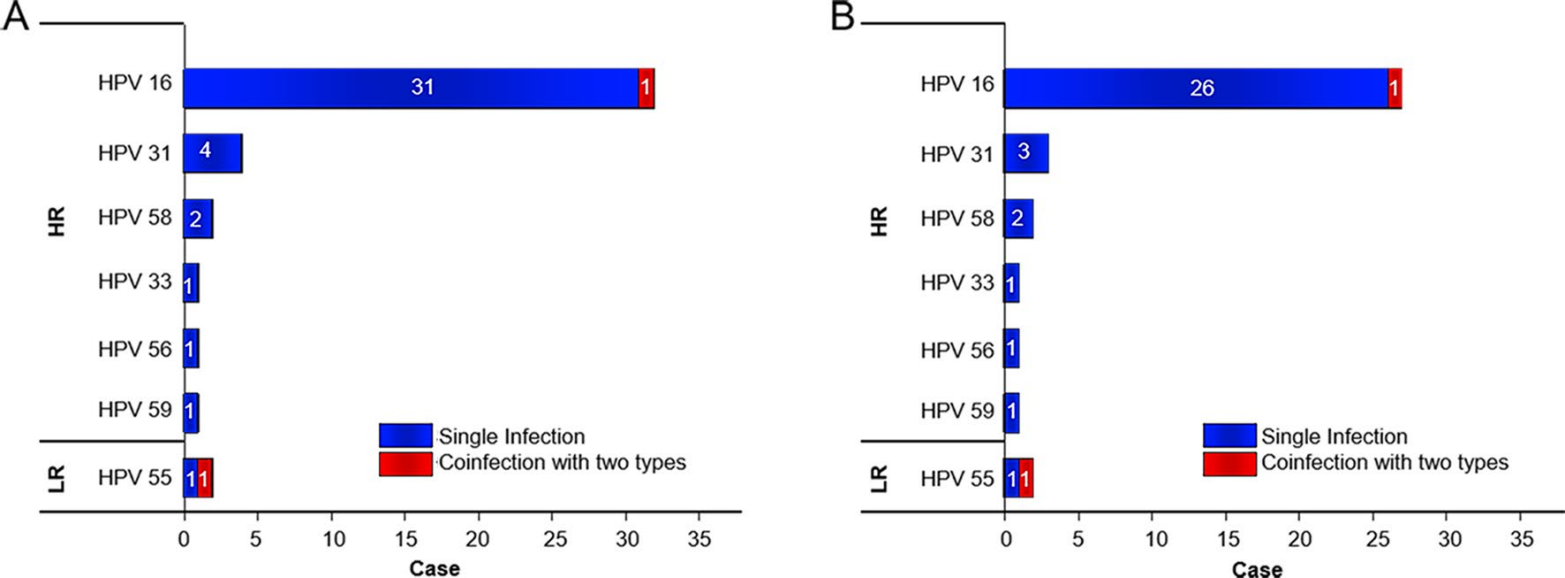
**a** The distribution of HPV types in gynecologists who performed electrosurgery. **b** The distribution of HPV types in gynecologists who performed LEEP

As shown in Fig. 1a, the most common HPV genotypes in the electrosurgery group were HPV16 [(32/42, 76.19%) and (32/469, 6.82%)], followed by HPV31 [(4/42, 9.52%) and (4/469, 0.85%)], HPV58 [(2/42, 4.76%) and (2/469, 0.43%)], HPV55 [(2/42, 4.76%) and (2/469, 0.43%)], HPV33 [(1/42, 2.38%) and (1/469, 0.21%)], HPV56 [(1/42, 2.38%) and (1/469, 0.21%)] and HPV59 [(1/42, 2.38%) and (1/469, 0.21%)]. Among them, there were 41 cases of single HPV infection and 1 case of multiple infection with HPV16 and HPV55. In the group of participants who did not perform electrosurgery of the cervix, the types of HPV were HPV16 (3/231, 1.3%) and HPV43 (1/231, 0.4%).

As shown in Fig. 1b, HPV16 [(27/37, 72.97%) and (27/356, 7.58%)] was the most prevalent HPV subtype among participants performing LEEP, followed by HPV31 [(3/37, 8.11%) and (3/356, 0.84%)], HPV58 [(2/37, 5.41%) and (2/356, 0.56%)], HPV55 [(2/37, 5.41%) and (2/356, 0.56%)], HPV33 [(1/37, 2.70%) and (1/356, 0.28%)], HPV56 [(1/37, 2.70%) and (1/356, 0.28%)] and HPV59 [(1/37, 2.70%) and (1/356, 0.28%)]. Among them, there was one coinfection with both HPV16 and HPV55 in the LEEP group. All HPV genotypes were high-risk, except HPV55.

### Correlation between risk factors and HPV-positive rates among performing electrosurgery of the cervix

The possible risk factors for HPV positivity of operator nasal swabs were analyzed. As shown in Table 5, most (43.07%, 202/469) of the participants who performed electrosurgery (including LEEP) were 30–40 years of age. According to the results, older doctors (above 50 years of age) had the highest HPV infection rate in nasal swabs. However, the association between age and the presence of HPV in our participants was not statistically significant (*P *= 0.083). In addition, whether an increasing duration of electrosurgery causes higher HPV infection rates was investigated. Participants who had performed electrosurgery for more than 15 years were more prone to HPV infection (17.33%, 13/75) than those who had performed electrosurgery for 0–5 years (6.15%, 15/244), 5–10 years (7.22%, 7/97) and 10–15 years (13.21%, 7/53). This result indicated that there was a significantly higher HPV risk in doctors who had performed electrosurgery for a longer period (*P* < 0.001).

**Table 5:** The association between HPV detection rates and participants who operate electrosurgery according to age, operation time of electrosurgery, mask, smoke absorbing device and risk consciousness

Factor	HPV testing in nasal swabs		Total	*χ*^2^	*P* value
	Positive	Negative			
Age (years)				6.689	0.083
21–29	8 (5.84%)	129 (94.16%)	137		
30–39	21 (10.40%)	181 (89.60%)	202		
40–49	7 (7.07%)	92 (92.93%)	99		
≥ 50	6 (19.35%)	25 (80.65%)	31		
Operation time of electrosurgery (years)				10.351	0.016
0 ≤ *t* < 5	15 (6.15%)	229 (93.85%)	244		
5 ≤ *t* < 10	7 (7.22%)	90 (92.78%)	97		
10 ≤ *t* < 15	7 (13.21%)	46 (86.79%)	53		
≥ 15	13 (17.33%)	62 (82.67%)	75		
Surgical mask				37.315	0.000
No	9 (24.32%)	28 (75.68%)	37		
General mask	33 (13.98%)	203 (86.02%)	236		
N95 mask	0 (0%)	196 (100%)	196		
Smoke absorbing device				0.678	0.410
Yes	30 (9.74%)	278 (90.26%)	308		
No	12 (7.45%)	149 (92.55%)	161		
Risk consciousness				0.279	0.642
Yes	40 (8.83%)	413 (91.17%)	453		
No	2 (12.50%)	14 (87.50%)	16		

As shown in Table 6, the majority of participants performing LEEP were aged 30–40 years (44.94%, 160/356). The highest HPV-positive rate in nasal swabs was found in LEEP groups aged 30–40 (12.50%, 20/160) and over the age of 50 (12.50%, 3/24). However, there was no statistically significant relationship between age and HPV infection (*P *= 0.44). Most LEEP operators had over 15 years of LEEP experience. The HPV-positive rate was significantly higher in the group who had longer electrical surgery times with LEEP (*P *= 0.017).

**Table 6:** The association between HPV detection rates and gynecologists operating LEEP according to age, operation time of electrosurgery, mask, smoke absorbing device and risk consciousness

Factor	HPV testing in nasal swabs		Total	*χ*^2^	*P* value
	Positive	Negative			
Age (years)				2.703	0.44
21–29	6 (6.38%)	88 (93.62%)	94		
30–39	20 (12.50%)	140 (87.50%)	160		
40–49	7 (8.97%)	71 (91.03%)	78		
≥ 50	3 (12.50%)	21 (87.50%)	24		
Operation time of electrosurgery (years)				10.149	0.017
0 ≤ *t* < 5	12 (6.94%)	173 (93.51%)	185		
5 ≤ *t* < 10	5 (7.94%)	58 (92.06%)	63		
10 ≤ *t* < 15	7 (15.22%)	39 (84.78%)	46		
≥ 15	12 (19.35%)	50 (80.65%)	62		
Surgical mask				30.891	0.000
No	7 (25.00%)	21 (75.00%)	28		
General mask	29 (16.20%)	150 (83.80%)	179		
N95 mask	0 (0%)	149 (100%)	149		
Smoke absorbing device				0.283	0.713
Yes	25 (10.73%)	208 (89.27%)	233		
No	11 (8.94%)	112 (91.06%)	123		
Risk consciousness				0.279	0.642
Yes	34 (9.94%)	308 (90.06%)	342		
No	2 (14.29%)	12 (85.71%)	14		

Additionally, the most common protective measures during electrosurgery of the cervix included surgical masks and smoke suction devices. In our research, 432 doctors wore a protective mask during electrosurgery, and the remaining 37 participants (7.89%, 37/469) wore no surgical mask during the operation. The general mask was the most common protective mask (54.63%, 236/432), followed by the N95 mask (45.37%, 196/432). The HPV-positive rate was significantly lower in cervical electrosurgery operators who used a surgical mask (7.64%, 33/432) than in those who did not use a protective mask (19.15%, 9/37). Furthermore, use of the N95 mask in electrosurgery (0%, 0/196) significantly reduced the risk for HPV infection compared to that with the general mask (13.98%, 33/236, *P *< 0.001). As shown in Table 6, a similar result was demonstrated in the LEEP operator group. The HPV-positive rate was significantly decreased in doctors who wore the N95 mask during LEEP (0%, 0/149, *P *< 0.001). Moreover, the rate of HPV detection in LEEP surgeons who used surgical masks (25.00%, 7/28) was remarkably higher than that in surgeons who did not wear a mask during LEEP (8.84%, 29/328).

Smoke absorbing devices were more commonly applied in electrosurgery (65.67%, 308/469). The use of smoke evacuation systems decreased the risk of HPV infection, but this difference had no statistical significance (*P *= 0.410). The other variable evaluated in this study was the risk consciousness pertaining to surgical smoke. Most of the participants (96.59%, 453/469) were aware of the hazards posed by surgical smoke to electrosurgery operators. However, the association between safety consciousness and rates of HPV positivity was not statistically significant (*P *= 0.642). As shown in Table 6, most of the participants who performed LEEP used smoke suction devices (65.45%, 233/356) and attached great importance to the hazards of surgical smoke (96.07%, 342/356). However, there was no significant correlation between suction device, risk consciousness and HPV infection in the LEEP group (*P *= 0.713, 0.642, respectively).

### Follow-up studies

All the surgeons in whom HPV DNA was detected were inspected again in the following 3 months, and the HPV DNA results in 20 doctors (43.48%, 20/46) who had been HPV positive in nasal swab samples turned negative. The other 26 doctors (56.52%, 26/46) still exhibited the same HPV genotypes in nasal swabs. Moreover, five participants who had a positive HPV test were withdrawn in the 24 months after the first survey. No HPV DNA could be detected in the remaining surgeons (0%, 0/41), which means that 100% (41/41) became negative for HPV DNA. This result indicated that the nasal HPV infection of surgeons was transient and not persistent. This may be because electrosurgery, including LEEP, caused inactivation of the HPV DNA in the surgical smoke. Therefore, although HPV DNA from surgical smoke was detected, it could not lead directly to HPV-associated diseases.

Thirty-six gynecologists who performed LEEP and had a positive HPV test in nasal swabs were followed up. Approximately 47.22% (17/36) became negative for HPV DNA, and 52.78% (19/36) still exhibited the same HPV genotypes in nasal swabs in the 3 months after the first survey. One gynecologist was lost to follow-up at 24 months because of departure from the hospital, and 100% (35/35) of the other gynecologists became negative for HPV DNA.

During the 24-month follow-up period, no HPV-related cancers or diseases, such as nasopharyngeal condyloma acuminatum, nasopharyngeal papilloma, and nasopharyngeal cancer, were found.

## Discussion

In the current study, we found that (1) gynecologists who performed electrosurgery operations, including LEEP for cervical lesions, especially those who were exposed to surgical smoke for a long time, were at risk for HPV-DNA infection and that (2) surgical masks, especially the N95 mask, effectively decreased the hazard of acquiring HPV DNA from surgical smoke exposure during cervical electrosurgery, including LEEP.

Electrosurgery (including electrocautery, laser ablation and LEEP) is one of the most common techniques in the diagnosis and treatment of gynecological diseases, such as various cervical lesions (Smith et al. 2017). Furthermore, HPV infection always results in various cervical lesions, including cervicitis, CIN I–III, and even cervical cancer. However, multiple problems are still posed by surgical smoke generated by electrosurgical units used for gynecological diseases. The potential for HPV viral transmission through surgical smoke has been shown by various authors. First, Hallmo and Naess (1991) demonstrated HPV-related diseases, such as papillomatosis, in surgeons who perform electrosurgery to treat HPV-relevant lesions. Furthermore, Rioux et al. (2013) reported that a 53-year-old male gynecologist developed HPV 16 positive tonsil squamous cell carcinoma, who had operated LEEP treatment for over 20 years on cervical and vulvar lesions. These reports confirm that our perioperative personnel could be infected with HPV DNA and further suffer related cancers caused by long-term inhalation of viral particles present in surgical smoke.

In recent decades, some studies have suggested a low risk of HPV transmission to surgeons during operations (Ferenczy et al. 1990). Weyandt et al. (2011) found that the HPV type in swabs of the nasolabial folds of one physician performing CO_2_ laser ablation to treat genital warts was HPV 38 before and after treatment, which different from that in the patients, who were positive for HPV6 and HPV11. HPV DNA in doctors most likely originated from the physicians themselves.

However, some researchers have tried to verify the presence of papillomavirus DNA in surgical smoke. The most frequently cited research demonstrating HPV infectiousness was from Garden et al. (2002), who first detected bovine papillomavirus DNA in surgical smoke. Sawchuk et al. (1989) included a group of human and bovine warts that were treated with carbon dioxide laser or electrocoagulation, and collected the smoke plume produced by each form of therapy. The researchers found that surgical masks could effectively block almost all laser- or electrocoagulation-derived viruses. However, only a few cases were included in this study.

In reality, most of the studies about the correlation between surgical smoke produced from electrosurgery and HPV infection have been case reports or studies with a small size. Our study included 67 hospitals comprising a variety of municipal and township hospitals, spread all over Zhejiang Province, and demonstrated that HPV DNA existing in surgical smoke generated from LEEP or other electrosurgeries on the cervix may lead to nasal HPV infection of gynecologists. The prevalence and genotype distribution of HPV in our study was basically consistent with the common HPV genotype types of cervical lesions in Zhejiang Province.

At present, HPV infection is defined as an existing and moderate risk to surgeons in European Union countries (Neumann et al. 2018). Therefore, it is of considerable importance to protect gynecologists from the inhalation of HPV aerosols using effective preventive measures such as surgical masks and suction devices. Recently, N95 surgical mask respirators were found to offer considerably improved protection over that provided by surgical masks (Edwards and Reiman 2012). In our present study, the rate of HPV positivity in the group who wore masks (including general or N95 masks) was significantly lower than that in the control group who did not wear any masks. Additionally, no HPV DNA was identified in electrosurgery operators who wore N95 masks, which suggested that the N95 mask could effectively prevent HPV viral infection to surgeons during electrosurgery, including LEEP. Furthermore, the use of a smoke evacuation system was another effective protective measure (Wang et al. 2015). In addition, general operation room ventilation, which uses central smoke-evacuation systems, is also insufficient to effectively capture smoke generated at the surgical site (Springer 2007). In our research, the use of a smoke evacuation system reduced the HPV incidence compared to that in the group without any smoke evacuation systems. However, there was no significant difference. Our study demonstrated that the rate of HPV DNA detection in operators’ nasal swabs was significantly increased in electrosurgical procedures, including LEEP, and the HPV-positive rate in surgeons wearing surgical masks was significantly decreased.

## Conclusions

In conclusion, this study provides further evidence to suggest that surgical smoke produced from electrical operations, including LEEP, poses the risk of contamination with HPV virus. It is of great importance for operators to use effective measures, such as high-filtration masks and smoke evacuation systems, to avoid HPV infection when performing surgery. Moreover, surgeons and perioperative personnel should strengthen protective awareness regarding the potential health risks associated with long-term exposure to surgical smoke.

## Electronic supplementary material

Below is the link to the electronic supplementary material.

Supplementary material 1 (DOCX 15 kb)
