# Supplementary material for: Prevalence of HPV infections in surgical smoke exposed gynecologists
Source: Int Arch Occup Environ Health. 2020 Sep 1;94(1):107–15. doi: 10.1007/s00420-020-01568-9 (PMC7826298; doi:10.1007/s00420-020-01568-9)
Supplement: Supplementary file 1 — Supplementary material 1 (DOCX 15 kb) [file 420_2020_1568_MOESM1_ESM.docx]

Supplementary Materials

for

**Prevalence of HPV infections in surgical smoke exposed gynecologists**

Xiaoli Hu ^#^, Qingfeng Zhou ^#^, Jian Yu, Jing Wang, Quanmei Tu, Xueqiong Zhu *

*Department of Obstetrics and Gynecology, the Second Affiliated Hospital of Wenzhou Medical University, Wenzhou 325027, China.*

***Corresponding author**: Xueqiong Zhu

*Mailing address:* No. 109 Xueyuan Xi Road, Department of Obstetrics and Gynecology, the Second Affiliated Hospital of Wenzhou Medical University, Wenzhou, Zhejiang, 325027, China.

Tel: +86 577 88002796; Fax: +86 577 88832693.

*E-mail address:* [zjwzzxq@163.com](mailto:zjwzzxq@163.com).

^#^ These authors contributed equally to this work.

**Questionnaire of the relationship between LEEP and HPV positive rates in medical staff’s nasal pharynx**

Dear friends, thanks for your help. Our group is from the department of obstetrics and gynecology in the second affiliated hospital and Yuying children’s hospital of Wenzhou medical university.

Several studies showed that HPV existed in surgical smoke produced from loop electrosurgical excision procedure (LEEP), which could cause the high-risk HPV virus infection in medical workers’ nasopharynx, and might lead to related lesions. Whether you have a LEEP operation or not, we will take a nasal swab sample for you to detect the HPV virus. The testing methods included polymerase chain reactions (PCR) and hybridization, which could detect 26 HPV types and 6 high-risk HPV types, respectively. If the test result was positive, we will inform you as soon as possible. Meanwhile, you can get the free follow-up test. All of the tests were free, and we will guarantee you that your answers and related results will be kept strictly confidential. Thank you very much for your support and cooperation.

Number: ___________ Hospital: _____________

1. Your name: ____________

2. Your age: _____________

3. Your telephone number: _______________

4. Have you made the electrical surgery? A. Yes B. No

5. What is your type of electrical operation? __________

A. LEEP B. Electrocautery C. Both D. None

6. How long time did you perform the electrical surgery (year)? ____________

A. 0-5 B. 5-10 C. 10-15 D. ≥ 15

7. Did you take any protective measure during the electrical surgery? ____________

A. Yes B. No (Please continue your answer if you choose option A)

8. Did you wear a mask when you make the electrical operation? ______________

A. No B. General mask C. N95 mask D. General mask + N95 mask

9. Did you use the smoke absorbing device when you make the electrical operation?

A. No B. Electric suction pump C. Central attractor D. Electric + central suction pump

10. Did you think that the surgical smoke produced from LEEP was harmful to people?

A. Yes B. No

I agree to participate in this investigation, and cooperate with sampling the nasal swab for high-risk HPV detection.

Please your name and the date: _______________________________________
